# Evaluation of the effect of vitamin D3 on mandibular condyles in an ovariectomized mouse model: a micro-CT study

**DOI:** 10.1186/s12903-021-01980-8

**Published:** 2021-12-07

**Authors:** Szandra Körmendi, Bálint Vecsei, Szilvia Ambrus, Kaan Orhan, Csaba Dobó-Nagy

**Affiliations:** 1 Department of Prosthodontics, Semmelweis University, Szentkirályi u. 47, Budapest, 1088 Hungary; 2 Department of Dentomaxillofacial Radiology, Faculty of Dentistry, Ankara University, Emniyet Mah.İncitaş sokak.Sabancı Kız yurdu karşısı, 06500 Ankara, Turkey; 3 Department of Oral Diagnostics, Semmelweis University, Szentkirályi u. 47, Budapest, 1088 Hungary

**Keywords:** Osteoporosis, Micro-CT, Condyle, Vitamin D3

## Abstract

**Background:**

This study aimed to investigate the effect of ovariectomy and vitamin D3 on bone microstructure; this effect was examined in three regions of interest at one femoral and two mandibular sampling sites bone in an ovariectomized mouse model.

**Methods:**

Thirty-six week-old female mice were randomly divided into three groups: 10 subjects were given oral cholecalciferol (vitamin D3) daily for 6 weeks after undergoing bilateral ovariectomy (D3 group), while 10 ovariectomized subjects (OVX) and 10 subjects who underwent a sham operation (SHAM) received peanut oil daily during the investigation. After extermination, the left hemimandible and femur were removed and scanned by micro-CT. The bone micromorphology parameters were analyzed and the BMD was calculated.

**Results:**

The bone volume fraction (BV/TV) was significantly lower in the trabecular bone of the mandibular condyle in the OVX group than in the SHAM and D3 groups. Also there was a significant difference between the SHAM and D3 groups. The specific bone surface (BS/BV) was significantly higher in the OVX and D3 groups than in the SHAM group. Trabecular thickness (Tb.Th) was significantly higher in the SHAM group, and the trabecular bone pattern factor (Tb.Pf) was significantly higher in the OVX group than in the other two groups. Bone mineral density (BMD) of the femur and the mandible was significantly lower in the OVX group than in the SHAM and D3 groups.

**Conclusions:**

Our results show that ovariectomy causes a significantly weaker bone microstructure in the mandibular condyle, where the protective effect of vitamin D3 resulted in a partial resorption.

## Background

Osteoporosis affects 200 million people worldwide, and this number is expected to increase dramatically in the future [1]. Most osteoporosis patients suffer from postmenopausal osteoporosis, but we have also seen an increasing number of other types of osteoporosis, particularly secondary osteoporosis [1]. In the prevention and therapy of the disease, adequate lifestyle and physical activity play a major role. Additional therapy may be anabolic or antiresorptive, depending on the type and severity of osteoporosis. Almost all of these treatments include vitamin D and calcium supplementation. Bone micromorphological changes in osteoporosis are different in cortical and trabecular bone, and vary in different bones. Analyzing bone microarchitecture changes with micro-CT is a non-destructive, fast and reliable method, also the adjustable resolution provides a great way to examine small rodents as well as larger species [2]. While vertebra, femur and tibia studies in osteoporotic rodent models show cortical narrowing and weakened trabecular structure, the results of examinations of the mandible tend to diverge [3]. Several authors suggest that this is partly because the regions of interest (ROIs) in the femur, vertebra and tibia are determined in a well-defined and identical place, while the ROIs in the mandible and the maxilla may vary significantly [3, 4]. A frequently used mandibular sampling site is the intermolar spongiosa and—for cortical examination—the base of the mandible. In subject with osteoporosis, micromorphological changes in the jaw can significantly influence the progression of periodontal disease or the success of osseointegration around an implant [5]. Further areas of interest are the mandibular condyle and the temporomandibular joint (TMJ). According to some studies, people with osteoporosis have a higher incidence of temporomandibular dysfunction [6, 7], while, according to other studies, there is no correlation in the simultaneous occurrence of osteoporosis and temporomandibular dysfunction (TMD) [8]. The effects of drugs used in osteoporosis therapy on the mandibular condyle and condylar cartilage are increasingly under scrutiny [9].

The investigation of bone microstructure changes following ovariectomy in the condyle is very important, especially when examined in conjunction with the more widely used sampling sites. There is limited literature on this matter.

This study aimed to investigate the changes in bone microstructure at two mandibular sampling sites together with femoral BMD in the ovariectomized mouse model and to examine the influence of vitamin D3 administration on the effect of ovariectomy.

## Methods

Thirty-six week-old, 22 g CRL: OF1 (Charles River Laboratories) female mice were randomly divided into three equal groups: 10 subjects were given oral cholecalciferol (vitamin D3) (4 ng/day Alpha D3-TEVA 0.25 µg) daily for 6 weeks after undergoing bilateral ovariectomy (D3 group), while 10 ovariectomized subjects (OVX group) and 10 subjects after a sham operation (SHAM group) were administered peanut oil (the carrier used for vitamin D) daily for 6 weeks during the investigation. Mice in the D3 group were administered vitamin D (Alpha D3 TEVA 0.25) orally, dissolved in peanut oil, mice in the SHAM and OVX groups were administered the same amount of peanut oil only. Peanut oil was administered in all groups orally by drip at the same time each day for 6 weeks starting on the first day after the ovariectomy or the SHAM operation. The unit amount of a standard pharmacy drop is equivalent to 0.024 g in the case of peanut oil. Administration of vitamin D3 began the day after surgery. The animals were kept in light controlled, air-conditioned rooms and allowed to eat and drink ad libitum. The diet of the mice was adjusted to include 0.025 mg cholecalciferol/kg, as recommended [10]. The animals were sacrificed by inhalation of 100% carbon dioxide. The concentration of carbon dioxide gradually increased with a displacement rate equivalent to 20% chamber volume per minute for 3 min. The Animal Experimentation Ethics Committee of Semmelweis University approved the care and experimental protocol of this study (22.1/2756/003/2007). After extermination, the left hemimandible and left femur were removed and stored in phosphate-buffered saline, containing 0.02% sodium azide at 4 °C. Scanning was performed on Skyscan 1172 (Bruker, Kontich, Belgium). The scanning protocol was set at X-ray energy, with settings of 50 kV and 198 µA, and the voxel size was 5.02 µm for the examination of the femur. The settings for the mandible base were 70 kV, 114 µA and 7.1 µm; the settings for the condyle were 60 kV, 156 µA and 3.9 µm. In all cases, there was a 0.5 mm Al-filter and a rotation degree of 0.5. On the femur, the starting slice of the ROI was at a distance of 50 slices (0.225 mm) from the growth plate at the distal epiphysis, in the direction of the diaphysis. From here, BMD was measured through 400 slices (1.807 mm). In the mandibular samples, the area between the roots of the first molar tooth was marked for BMD examination of the mandibular spongiosa. In the investigation of the condyle, the ROI was established by selecting all the trabecular bone in the mandibular condyle. When determining BMD, Bruker-MicroCT BMD calibration phantoms were used, with calcium hydroxyapatite (CaHA) concentrations of 0.25 and 0.75 g/cm^3^. In line with the Bruker-MicroCT recommendation, we chose a pair of 2 mm diameter rods. Following the density range calibration to air and water the following equation was used in the BMD calculation:

$$BMD = \frac{124,961805 - HU}{{4912,70746}}\left( {\frac{{\text{g}}}{{{\text{cm}}^{3} }}} \right)$$

It was important to use all the decimal places to optimize the accuracy of the calibration.

Reconstruction was performed with the NRECON (Skyscan, Bruker) software, and analyses of the microarchitecture of the mandible and the femur were implemented with CT Analyser 1.7.0.0. (Skyscan, Bruker) software. The global manual threshold technique was applied during segmentation. The lower threshold was 102 and the upper was 255. Bone volume fraction (BV/TV), specific bone surface (BS/BV), trabecular thickness (Tb.Th), trabecular bone pattern factor (Tb.Pf) and bone mineral density (BMD) values were determined in the samples. A decrease in BV/TV indicated a decrease in bone tissue in the sample, as did a decrease in Tb.Th, which indicates a thinning of the trabeculae. An increasing BS/BV value could be observed when resorption in bone tissue was  predominated. The Tb.Pf value was calculated and not measured; it increased as the number of connections between the trabeculae decreased, signalling a weaker microstructure or different microarchitecture [11]. Tb.Pf is a counted value by comparing the 2-dimensional area (A) and perimeter (P) or the 3-dimensional volume and surface of a binarized object before (A1 and P1) and after (A2 and P2) dilation of an image. The formula for calculation of Tb.Pf used by the CT Analyser 1.7.0.0. software (Skyscan, Bruker):  Tb.PF=(P1-P2)/(A1-A2).

Statistical analysis was performed using SPSS 24.0 software (SPSS, Chicago, IL, USA). The Kruskal–Wallis test was applied with significance set at *p* < 0.05, and then a Dunn post hoc test was used.

## Results

**Fig. 1:**
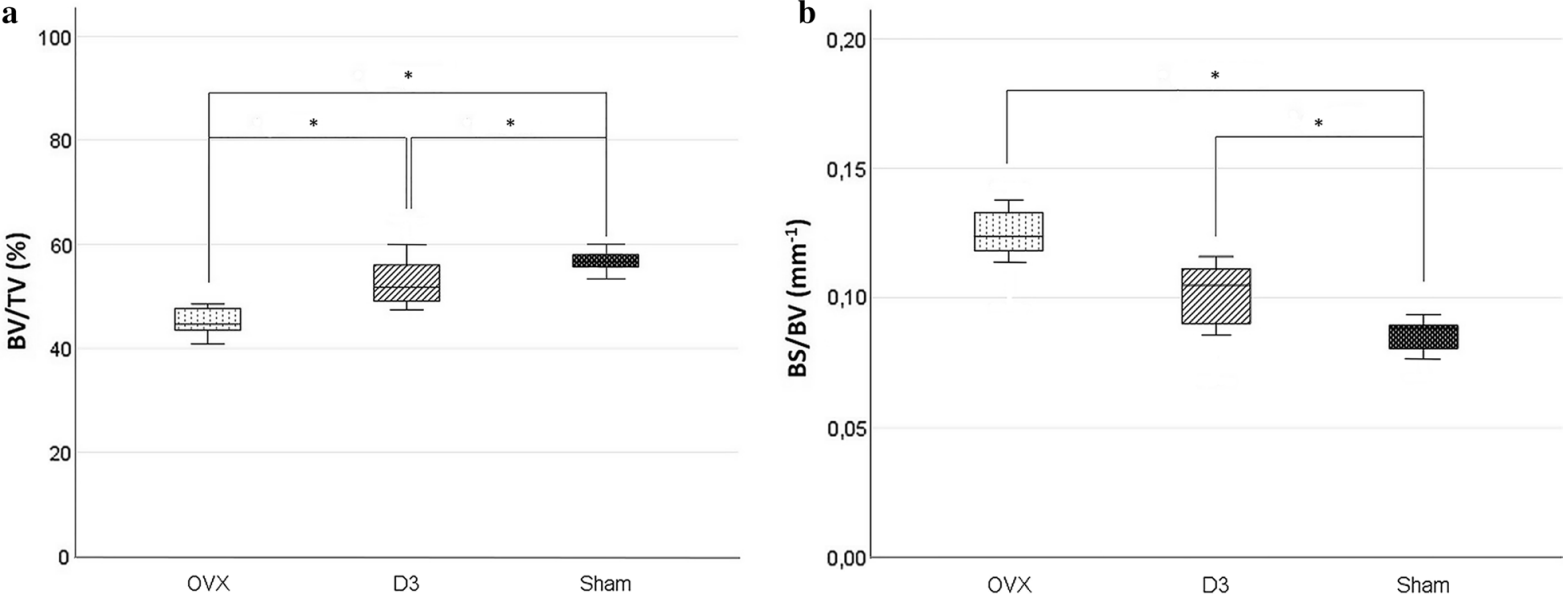
Bone volume fraction (BV/TV) (**a**) and specific bone surface (BS/BV) (**b**) results in mandibular condyle in OVX, D3 and SHAM groups

In the mandible, the BV/TV was significantly lower in the trabecular bone of the condyle in the OVX group (44.93% ± 4.09) than in the SHAM (57.61% ± 4.5) and D3 (51.47% ± 5.01) groups; also there was a significant difference between the SHAM and D3 groups. The bone BS/BV was significantly higher in the OVX group (0.125 mm^−1^ ± 0.015) and in the D3 group (0.109 mm^−1^ ± 0.016) than in the SHAM group (0.088 mm^−1^ ± 0.012) (Fig. 1).

**Fig. 2:**
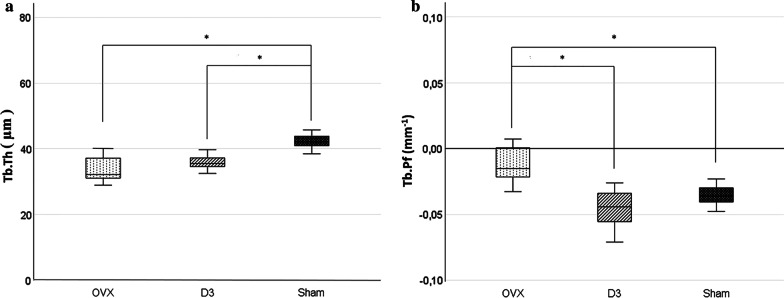
Trabecular thickness (Tb.Th) (**a**) and trabecular bone pattern factor (Tb.Pf) (**b**) in the mandibular condyle in OVX, D3, and SHAM groups

Tb.Th values were significantly higher in the SHAM group (42.8 µm ± 3.1) than in the OVX (34.42 µm ± 4.9) and D3 (36.23 µm ± 3.1) groups. The Tb.Pf values were significantly higher in the OVX group (− 0.01 mm^−1^ ± 0.003) than in the SHAM (− 0.039 mm^−1^ ± 0.01) and D3 (− 0.043 mm^−1^ ± 0.01) groups (Fig. 2).

**Fig. 3:**
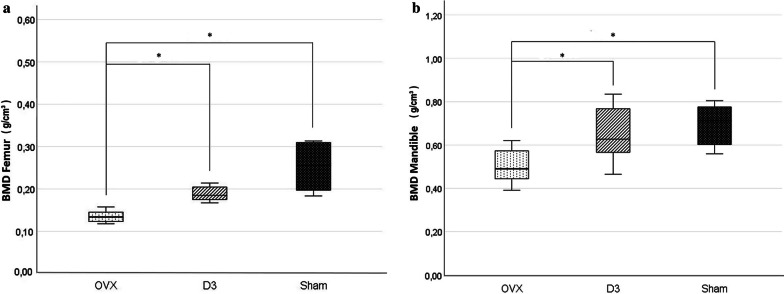
Bone mineral density (BMD) values of the femur (**a**) and mandible (**b**) in OVX, D3 and SHAM group

BMD values observed during the examination of the femur were significantly lower in the OVX group (0.14 g/cm^3^ ± 0.02) than in the SHAM (0.25 g/cm^3^ ± 0.05) and D3 (0.18 g/cm^3^ ± 0.03) groups. The results of the BMD examination of the mandible also showed significant differences: the values in the OVX group (0.5 g/cm^3^ ± 0.07) were also lower here than in the SHAM (0.69 g/cm^3^ ± 0.08) and D3 (0.64 g/cm^3^ ± 0.07) groups (Fig. 3).

## Discussion

For the micro-CT examination of long bones such as the femur or tibia, the use of a well-defined and large-volume ROI is possible [3]. In contrast, the mandibular condyle needs to be examined with a small volume ROI. Therefore, in the case of bone microarchitecture and especially BMD calculations, femur data can also be used as a control in case other examination sites are applied in the model as well.

The micromorphological parameters measured in our previous research are in line with the BMD results obtained for the femur in this study [12]. Significantly lower BV/TV and Tb.Th values were obtained in the OVX group compared to the SHAM group, and significantly higher Tb.Pf values were found in the OVX group compared to the SHAM group, under the same test conditions and at the same ROI site. These results suggest that ovariectomy leads to a significantly reduced bone structure. However, the D3 group did not differ significantly from the SHAM group, and the advantage of vitamin D3 is that there was no significant microarchitecture damage [12].

The BMD values of the mandibular spongiosa are similarly supported by the micromorphological analysis of our previous study [12]. With the same test conditions and the same ROI, the OVX group had significantly lower BV/TV and Tb.Th values than in the SHAM and D3 groups, also BS/BV values were higher in the OVX group than in the SHAM group. These results show that microstructure changes affect the mandible, and the protective effect of vitamin D3 was also present [12].

**Fig. 4:**
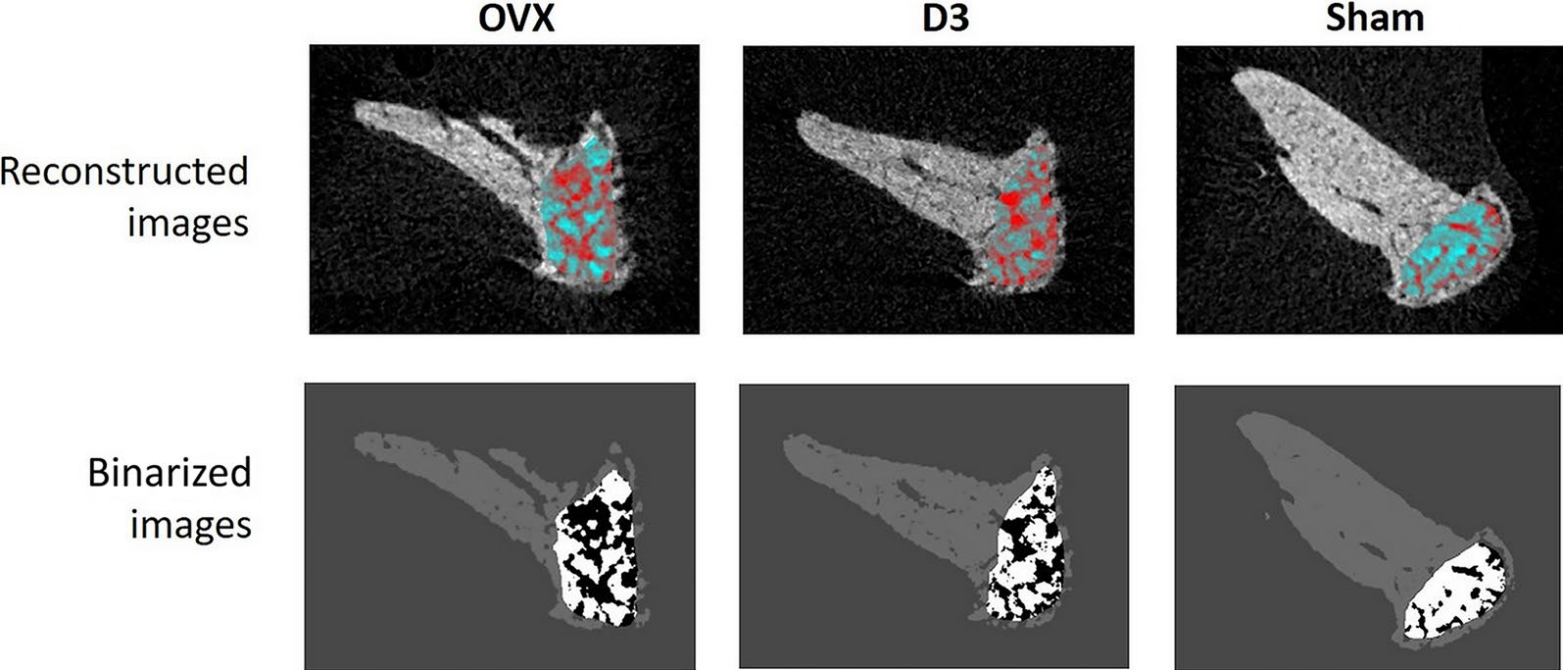
Reconstructed and binarized images of condyle with ROI in OVX, SHAM and D3 groups

The analysis of the mandibular condyle data indicated that BV/TV values were significantly lower and BS/BV values were significantly higher in the OVX group than in the SHAM group, so a weaker structure in this bone was also caused by ovariectomy. The differences in BV/TV and BS/BV parameters between the D3 group and the SHAM group, may indicate that the protective effect of D3 on bone structure is not completely expressed in this area. The significant increase in Tb.Pf in the OVX group compared to the SHAM group shows a decreased number of connections between the trabeculae, which is a sign of a significant weakening of the microarchitecture. In accordance with non-linear pattern formation (NPF), resorptions and formations in the bone cause a dynamic change in the microstructure with a non-linear pattern, so quantitative changes in some morphological data do not necessarily reflect the real architecture of bone [13]. Based on the NPF concept, micromorphological analysis is particularly difficult for small volume samples, and special attention should be given to the selection of parameters [13] (Fig. 4).

Knowing the effect of osteoporosis or its treatment is important for practitioners for many reasons. Areas of particular interest are the alveolar bone and TMJ. As early as 1995, Klemetti et al. suggested that patients with osteoporosis may have an increased incidence of craniomandibular dysfunction (CMD) [14]. Some studies suggest that not only the microstructure of the jaws but also the condylar cartilage may be affected by decreased oestrogen levels in patients with postmenopausal osteoporosis and possibly by the use of bisphosphonate in osteoporosis therapy [9, 15].

Studies published two decades earlier showed that ovariectomy resulted in significant minor changes in the mandibular condyle [16, 17] or the alveolar process of the mandible [18] compared to long bones. According to some osteoporosis studies, attenuation of the trabecular structure of long bones could not be detected in the alveolar or interradicular areas of the mandible. This discrepancy is often interpreted to be the result of mechanical stimuli stemming from the constant occlusal load of the teeth. Nevertheless, inflammation with an endodontal or periodontal origin can cause increased bone loss unrelated to osteoporosis [4]. This also makes it difficult to select an ROI in jaw bones due to the small size and complex geometry. Some research also shows that the area selection of the ROI can lead to considerable measurement differences, not only in the mandible. In the case of the vertebra, ROI selection can also have an important effect on the BMD and microstructural changes depending on the craniocaudal dimension [19]. Studies also confirm that normal (hard) consistency food, also used in this study, may significantly limit the negative effects of oestrogen deficiency on the alveolar bone structure of the mandible in a rat model [20].

In more recent studies, using more sophisticated software, in the alveolar trabecular bone of the mandible and in the condyle, which is similar to the femur, significantly weaker bone structure was present after ovariectomy [21–23]. This was also shown in our study. In their research, Liu et al. compared micromorphological data from different sampling sites of the mandible and the tibia in an ovariectomized mouse model. They found that the data of the tibia strongly correlated with the data of the spongiosa between the roots of the first molar, while values around the second and third molars were not correlated with this data [21]. This result influenced our choice of the sampling area.

Another study also examined the microarchitecture of bone tissue around the first molar of the mandible and the tibia in an ovariectomized rat model, and there was a significant correlation among BS/BV, Tb.Th and Tb.Sp values [23]. Yang et al. found a significant positive correlation in an ovariectomized rat model between Tb.Sp and SMI parameters when comparing microstructural data of the mandible and the tibia [24].

Kosugi et al. compared the micromorphological changes in the femur and condyle, and they found significant microarchitectural damage in subjects with osteoporosis [25], which is congruent with our findings.

In an ovariectomized mouse model, Hao et al. found a significant reduction in BMD only in long bones, and the micromorphological values reflected weaker bone; however the BMD of the mandible did not follow the micromorphological parameters of this area [22]. In the study of Liu H et al., the BMD values showed no correlation between the mandible and the tibia [21]. We found contradictory results, e.g. mandibular BMD followed the changes of micromorphological values. The difference between the results of previous studies and those of our studies could be due to our use of preliminary calibration of BMD [21] and the references used for calibration [22]. The geometry of the rod phantoms used for BMD calibration is also important since there is an exponential relation with their diameter.

Changes in BMD in the femur and the mandible were also studied by Hao's research group, and significant reductions were found in both areas in the OVX group compared to the SHAM group [26]. Our results also support this outcome.

Liu et al. used an ovariectomized rat model to compare the micromorphological characteristics by time in several bones. Their results showed that long bones, lumbar vertebra and ilium had a similar trend of bone loss after ovariectomy, although the authors found the highest bone loss in the femur and tibia, while the spine seemed to be the least sensitive during the 36-week examination. Micromorphological data (Tb.Sp, Tb.Th, and BV/TV) also showed that while data from the femur and tibia were significantly different between the groups at 2–4 weeks, in the case of the mandible and maxilla, the greatest difference was observed at weeks 24 and 36, and the mandible showed a greater response than the maxilla in the OVX group [27]. Mouse studies showed a similar temporal relationship [28]; therefore in our study, we followed the experimental period of previous studies.

Lee et al.'s systematic review and meta-analysis yielded the following results: in the OVX group, BV/TV, Tb.Th and BMD values in the selected ROI in the mandible displayed bone loss consistent with osteoporosis compared to the control group. Tb.Sp values increased in the OVX group. However, Tb.N values showed no indication of osteoporosis. Meta-regression analysis indicated that a longer (exposure) time after ovariectomy leads to greater changes in BMD. According to the authors, the heterogeneity of the data is mainly due to the different lengths of the post-procedure period in the OVX group and the different ROIs evaluated [3].

Our micromorphological data of the condyle suggest that a partial resorption is present in the D3 group, but this is far from the same as in the OVX group. There is no significant difference between the OVX and D3 groups for BS/BV and Tb.Th values. BS/BV values show that the bone surface became more eroded and the trabeculae thinned. It is at the crosslinks that trabecular resorption was the least observed (based on Tb.Pf values). This pattern of change is biomechanically advantageous.

It needs to be explained why vitamin D3 has only a partial protective effect in the area of the condyle, in contrast to the long tubular bones or the interradicular areas of the mandible, even though vitamin D3 was administered from zero day of the experiment. The reason why partial protective effect of vitamin D3 on the mandibular condyle was only observed in the present study requires further clarification. We do not believe that increasing the dose of vitamin D3 would result in a protective response in this area, as the administration of vitamin D3 was based on supplementary therapeutic doses. During the development of condyles, endochondral ossification occurs, while in the rest of the mandible, the ossification is intramembranous [29]. Perhaps this also plays a role in the different treatment responses in this area in addition to the completely unique mechanical load of the condyles. This can be explained by the slower bone remodeling rate compared to other areas of the mandible [30]. Additional research is suggested at the molecular level, investigating the number of vitamin D receptors (VDRs) in the bone tissue of the condyle, which may be different from other areas of the mandible. This idea might support an observation where the VDR gene is associated with mandibular retrognathism [31]. However, there is no information available in the literature on the suggested difference in vitamin D receptor expression in this area. Further research is required to provide an explanation of this phenomenon.

## Conclusion

Our results show that ovariectomy causes a significantly weaker bone microstructure in the mouse mandibular condyle, where the protective effect of vitamin D3 resulted in a partial resorption. In the condyle vitamin D3 modulates the process of bone resorption, but similar protective effect than in long bones is not observed.

## Data Availability

The datasets used during the current study are available from the corresponding author on reasonable request.

## Abbreviations

ROI: Region of interest
TMJ: Temporomandibular joint
BMD: Bone mineral density
TMD: Temporomandibular dysfunction
CaHA: Calcium hydroxyapatite
HU: Hounsfield unit
BV/TV: Bone volume fraction
BS/BV: Specific bone surface
Tb.Th: Trabecular thickness
Tb.Pf: Trabecular bone pattern factor
Tb.N: Trabecular number
Tb.Sp: Trabecular spacing
CMD: Craniomandibular dysfunction
VDR: Vitamin D3 receptor
NPF: Non-linear pattern formation
